# Arginine Vasopressin Alters Both Spontaneous and Phase-Locked Synaptic Inputs to Airway Vagal Preganglionic Neuron via Activation of V_1a_ Receptor: Insights into Stress-Related Airway Vagal Excitation

**DOI:** 10.3389/fncel.2017.00012

**Published:** 2017-02-02

**Authors:** Xianxia Yan, Xingxin Chen, Yuhong Guo, Ding He, Yonghua Chen, Chunmei Xia, Jijiang Wang

**Affiliations:** ^1^Department of Neurobiology, School of Basic Medical Sciences, Fudan UniversityShanghai, China; ^2^Department of Physiology and Pathophysiology, School of Basic Medical Sciences, Fudan UniversityShanghai, China

**Keywords:** arginine vasopressin, airway vagal preganglionic neuron, stress, psychological, patch-clamp, asthma

## Abstract

The airway vagal preganglionic neurons (AVPNs) in the external formation of the nucleus ambiguus (eNA) play a major role in the vagal control of tracheobronchial smooth muscle tone and maintenance of airway resistance. The eNA receives vasopressinergic projection from the hypothalamic paraventricular nucleus (PVN), the key node for the genesis of psychological stress. Since airway vagal excitation is reportedly to be associated with the psychological stress-induced/exacerbated airway hyperresponsiveness in asthmatics, arginine vasopressin (AVP) might be involved in stress-related airway vagal excitation. However, this possibility has not been validated. This study aimed to test whether and how AVP regulates AVPNs. In rhythmically active medullary slices of newborn rats, retrogradely labeled AVPNs were identified as inspiratory-activated and inspiratory-inhibited AVPNs (IA- and II-AVPNs) using patch-clamp techniques according to their inspiratory-related firing behavior and synaptic activities. The results show that under current clamp, AVP depolarized both IA- and II-AVPNs, and significantly increased their spontaneous firing rate. Under voltage clamp, AVP elicited a slow inward current, and significantly increased the frequency of spontaneous excitatory postsynaptic currents (sEPSCs) in both types of AVPNs. In addition, AVP significantly enhanced the phase-locked excitatory inspiratory inward current in inspiratory-activated airway vagal preganglionic neurons (IA-AVPNs), but significantly suppressed the phase-locked inhibitory inspiratory outward current in II-AVPNs. In both types AVPNs, AVP significantly increased the frequency and amplitude of pharmacologically isolated spontaneous GABAergic and glycinergic inhibitory postsynaptic currents (IPSCs). All of the AVP-induced effects were prevented by SR49059, an antagonist of V_1a_ receptors, but unaffected by SSR149415, an antagonist of V_1b_ receptors. AVP did not cause significant changes in the miniature excitatory postsynaptic currents (mEPSCs), miniature inhibitory postsynaptic currents (mIPSCs) and membrane input resistance of either type of AVPNs. These results demonstrate that AVP, via activation of V_1a_ receptors, enhanced the spontaneous excitatory and inhibitory inputs similarly in the two types of AVPNs, but differentially altered their phase-locked inspiratory excitatory and inhibitory inputs. The overall effects of AVP are excitatory in both types AVPNs. These results suggest that increased central AVP release may be involved in the stress-induced augmentation of airway vagal activity, and, consequently, the induction or exacerbation of some airway diseases.

## Introduction

Airway vagal innervation originates principally from two sites in the brainstem: the nucleus ambiguus (NA) and dorsal motor nucleus of the vagus (DMNV; Jordan, 2001; Canning, 2006). Airway vagal preganglionic neurons (AVPNs) within these two regions serve as the final neural pathways transmitting signals from the central nervous system (CNS) to peripheral airways (Haxhiu et al., 2005). Noteworthily, a subset of AVPNs located in the external formation of the nucleus ambiguus (eNA) plays a predominant role in supplying cholinergic outflow to airway smooth muscles, and is crucial in regulating tracheobronchial caliber and airway resistance (Haselton et al., 1992; Canning and Fischer, 2001). According to their inspiratory-related electrophysiological properties *in vitro*, AVPNs in the eNA are classified into two categories: the inspiratory-activated and inspiratory-inhibited AVPNs (IA- and II-AVPNs; Haselton et al., 1992; Canning and Fischer, 2001). These AVPNs are intrinsically silent, and their spontaneous or phase-locked inspiratory activity depends completely on the balance between the excitatory, mainly glutamatergic, and the inhibitory, mainly γ-aminobutyric acidergic (GABA) and glycinergic, inputs that they receive. Alterations in this equilibrium are thought to be associated with dysregulation of pulmonary vagal functions (Haxhiu et al., 2005; Chen et al., 2007, 2012; Hou et al., 2012; Zhou et al., 2013; Ge et al., 2015).

AVPNs receive projections from multiple brain regions including the hypothalamic paraventricular nucleus (PVN), the key node for psychological stress to cause emotional and autonomic disorders (Haxhiu et al., 1993; Hadziefendic and Haxhiu, 1999; Schlenker et al., 2001; Herman et al., 2008; Ellenbogen et al., 2010); and a sense of causality has been prompted between psychosocial stress/negative emotions and augmentation of airway vagal activity (Isenberg et al., 1992; Ritz et al., 2010). As is well recognized, increased airway vagal tone is involved in the pathogenesis of airway hyperreactivity, the common traits of asthma (Barnes, 1992; Busse and Lemanske, 2001; Aboussafy et al., 2005; Ritz et al., 2012; McAlexander et al., 2015). Moreover, in asthmatics, bronchial hyperreactivity and the occurrence of bronchospasm can be induced or exacerbated by psychological stress, especially negative emotional stimuli (Heim et al., 1967; Put et al., 2004; von Leupoldt and Dahme, 2013). These lines of evidence indicate a functional connection between the PVN and the airway vagal centers, which may be involved in psychological stress-related asthma attack.

In psychological stress, arginine vasopressin (AVP) synthesized within the PVN is reported to be the initiator to trigger increased release of corticotrophin-releasing hormone (CRH) and subsequent activation of the hypothalamic-pituitary-adrenocortical (HPA) axis (Scaccianoce et al., 1991; Wotjak et al., 1996; Hatzinger et al., 2000); and the concentration of AVP is elevated in a stress level-dependent manner, in both plasma and cerebrospinal fluid (CSF; Bao et al., 2014). More precisely, AVP-containing fibers originating directly from the PVN are found just in the vicinity of AVPNs in the eNA (Kc et al., 2006). It has been widely accepted that AVP can act as a neuromodulator or transmitter mediating the alterations from central neuronal activities to psychological status (de Wied et al., 1993; Raggenbass, 2001; Keck, 2006). These underpinnings make it reasonable to think that vasopressinergic fibers may supply not only a morphological but also a functional connection between the PVN and AVPNs. However, currently it is unknown whether AVPNs express AVP receptors, and whether and how AVP regulates AVPNs, although a previous autoradiographic study found that the region dorsal to the NA shows positive binding to V_1_- receptor antagonist (Phillips et al., 1988). Thus this study aimed to uncover these issues at neuronal and at synaptic levels using patch clamp, by testing the hypotheses that AVP activates AVPNs directly and/or by regulating their synaptic inputs.

## Materials and Methods

### Animals and Ethical Approval

This study was carried out in accordance with the recommendations of the guidelines for the Care and Use of Laboratory Animals (1996. National Academy of Sciences, Washington, DC, USA). The protocol was approved by the Animal Ethics Committee of the Fudan University Shanghai Medical College (no. 20110307-060). Sprague–Dawley rats were purchased from the Experimental Animal Center of the Chinese Academy of Science in Shanghai. A total of 101 newborn rats of either gender were used in this study. Maximal efforts were made to minimize the number of animals and their suffering.

### Retrograde Fluorescent Labeling of AVPNs and Preparation of Medullary Slices

AVPNs were retrogradely labeled as we have previously described (Chen et al., 2007, 2012; Ge et al., 2015). In brief, the 2- to 3-day-old Sprague–Dawley rat was anesthetized with halothane and hypothermia. The extrathoracic trachea was exposed through a ventral midline incision in the neck, and the fluorescent tracer rhodamine (X-rhodamine-5- (and-6)-isothiocyanate; Molecular Probes, 1% solution, 0.5 μL) was injected into the smooth muscle at multiple sites of the anterior tracheal wall between the fourth and the eighth tracheal cartilage ring. The injection sites were sealed with absorbable gelatin sponge to prevent or absorb the possible leak of the dye. After a 48-h recovery, the rat was again deeply anesthetized and decapitated. The brainstem was isolated and submerged in cold (4°C) artificial cerebral spinal fluid (aCSF; mmol L^−1^: NaCl, 128; KCl, 3.0; NaHCO_3_, 24; NaH_2_PO_4_, 0.5; CaCl_2_, 1.5; MgCl_2_, 1; and D-glucose, 30) bubbled with 95% O_2_ and 5% CO_2,_ and a 500–800 μm thick medullary slice with rhythmic inspiratory-like hypoglossal bursting (Smith et al., 1991) was cut using a vibratome (VT 1000S; Leica Microsystems, Wetzlar, Germany). The slice was then transferred to a recording chamber (0.6 mL volume) with its rostral section upward, and superfused with high potassium (KCl, 8 mmol L^−1^; Smith et al., 1991) aCSF to allow steady recording of the rhythmic hypoglossal activity. The temperature of perfusate was maintained at 23 ± 0.5°C, and the flow rate was kept at 6–9 mL min^−1^.

### Electrophysiological Recording

The objective AVPNs were identified by the presence of fluorescence with an Olympus upright microscope (×40 water-immersion objective lens), as has been shown photographically in our previous works (Chen et al., 2007, 2012). Under infrared illumination, the identified AVPNs were imaged with an infrared-sensitive video detection camera to gain better spatial resolution and to visually guide and position the patch pipette onto these identified neurons. When recording the excitatory postsynaptic currents (EPSCs), the patch pipette (1.5–2.5 MΩ) was filled with a gluconate-K dominated solution (in mmol L^−1^: gluconate-K, 130; HEPES, 10; EGTA, 10; CaCl_2_, 0.1; 5′-ATP-K_2_, 2; MgCl_2_, 1; pH 7.3). The patch pipette was manipulated to slowly approach the identified AVPN to obtain a seal over 1 GΩ between the pipette tip and the cell membrane. The membrane sealing the pipette tip was then ruptured with a brief suction to gain a whole-cell patch-clamp configuration. The pipette resistance and capacitance were not compensated either before or after gaining intracellular access. Under voltage-clamp recordings, inspiratory-activated airway vagal preganglionic neurons (IA-AVPNs) were identified as those with the characteristic rhythmic inspiratory inward currents synchronous with the periodic inspiratory-like hypoglossal bursts. IA-AVPNs were normally clamped at −80 mV, and under this clamping voltage the inhibitory postsynaptic currents (IPSCs) caused by GABA and glycine are minimized. When putative AVPNs were clamped at −50 mV or more positive levels, II-AVPNs were distinguished by rhythmic inspiratory outward currents synchronous with the hypoglossal bursts. II-AVPNs were normally clamped at −50 mV, at which the glutamate-induced inward EPSCs and the GABA- or glycine-induced outward IPSCs are well separated. Under current clamp, IA-AVPNs exclusively exhibited rhythmic inspiratory depolarization and bursting discharge, while II-AVPNs displayed rhythmic inspiratory hyperpolarization. In some experiments that recorded miniature excitatory postsynaptic currents (mEPSCs), a 5-mV, 200-ms hyperpolarizing pulse was applied at a 4-s interval for calculation of input resistance.

In some experiments, to obtain better recordings of IPSCs, the patch pipette was filled with a KCl-dominated solution (in mmol L^−1^: KCl, 150; HEPES, 10; EGTA, 2; and ATP-Mg, 2, pH 7.3), and AVPNs were exclusively clamped at −80 mV. Under this configuration, all the synaptic currents including the glutamate-mediated inspiratory excitatory currents in IA-AVPNs and the GABA- or glycine-mediated inspiratory inhibitory currents in II-AVPNs were inward. In order to separate the two types of AVPNs from each other, the antagonists of glutamate receptors were focally implemented onto the recorded neurons with a puff pipette under the assistance of the PV830 Pneumatic Picopump pressure delivery system (World Precision Instruments, Sarasota, FL, USA). IA-AVPNs were then distinguished from II-AVPNs because their inspiratory inward currents were reversibly eliminated by the focally applied antagonists.

The patch-clamp signal was amplified with an Axon Multiclamp 700B amplifier (Molecular Devices LLC., CA, USA) with a sampling frequency of 10 kHz, and a filter frequency of 1 kHz. The activity of the hypoglossal rootlets was recorded through a suction electrode, amplified with a BMA-931 Bioamplifier (5 kHz sampling frequency, 10–1000 Hz bandpass, ×20,000 magnification; CWE, Ardmore, PA, USA) and electronically integrated (*τ* = 200 ms) with an MA-1000 Moving Averager (CWE). The patch-clamp signal and hypoglossal activity were digitized with a 1440A Digidata (Molecular Devices LLC., CA, USA), and collected with the Clampex 10.2 software (Molecular Devices LLC., CA, USA).

### Drug Application

Drugs were normally applied in the bath. AVP ([Arg^8^]-Vasopressin, Cys-Tyr-Phe-Gln-Asn-Cys-Pro-Arg-Gly-NH_2_) was normally used at 100 nM for 1 min. In some experiments, SR49059 (2S)-1-[[(2R, 3S)-5-Chloro-3-(2-chlorophenyl)-1-[(3, 4-dimethoxyphenyl)sulfonyl]-2,3-dihydro-3-hydroxy-1H-indol-2-yl]carbonyl]-2-pyrrolidinecarboxamide (20 μmol L^−1^), a potent and highly selective antagonist of vasopressin V_1a_ receptors (Serradeil-Le Gal et al., 1993), or SSR149415 (2S, 4R)-1-[(R)-5-Chloro-1-(2,4-dimethoxy-benzenesulfonyl)-3-(2-methoxy-phenyl)-2-oxo-2,3-dihydro-1H-indol-3-yl]-4-hydroxy-pyrrolidine-2-carboxylic acid dimethylamide (10 μmol L^−1^; Serradeil-Le Gal et al., 2003), a selective antagonist of vasopressin V_1b_ receptors (Serradeil-Le Gal et al., 2002), was applied at least 10 min prior to and during AVP application to block V_1a_ or V_1b_ receptors, respectively, and the subsequent AVP application was prolonged to at least 2 min. Strychnine (1 μmol L^−1^) and bicuculline (50 μmol L^−1^) were used to block glycine receptors and GABA_A_ receptors, respectively. CNQX (6-Cyano-7-nitroquinoxaline-2,3-dione; 50 μmol L^−1^) and AP5 (D-2-amino-5-phosphonovalerate; 50 μmol L^−1^) were used to block non-NMDA and NMDA glutamate receptors, respectively. When the neurons were recorded with the KCl-dominated pipette solution, CNQX and AP5 were first implemented focally to identify the type of the AVPN, and then included in the perfusate along with strychnine or bicuculline to isolate GABAergic or glycinergic IPSCs. Tetrodotoxin (TTX, 1 μmol L^−1^) was added to the perfusate in some experiments recording mEPSCs or miniature inhibitory postsynaptic currents (mIPSCs). SR49059, SSR149415, CNQX and bicuculline were firstly dissolved in DMSO and the other drugs in deionized water to make fresh stock solutions, and then diluted at least 1000 times with aCSF for experimental use. In each slice, only one AVPN was tested and each agonist or antagonist of vasopressin receptors was applied only once to minimize desensitization. AVP and SR49059 were purchased from Tocris Bioscience (Bristol, UK), SSR149415 was purchased from Axon Medchen (Groningen, Netherlands), and the rest of the drugs were purchased from Sigma-Aldrich (St Louis, MO, USA).

### Data Analysis

Spontaneous and miniature synaptic currents were analyzed with the MiniAnalysis software (version 4.3.1; Synaptosoft, Fort Lee, NJ, USA) with the minimal acceptable amplitude at 10 pA. When analyzing spontaneous excitatory postsynaptic currents (sEPSCs) occurred during the inspiratory intervals, the phase-locked inspiratory inward or outward currents were ignored. In each AVPN, a 1-min recording during control, AVP implementation, antagonist (SR49059 or SSR149415) application, or co-application of AVP and antagonist was analyzed for comparison. The rhythmic inspiratory inward/outward currents were analyzed with the Clampfit 10.2 software (Molecular Devices LLC.). Before analyzing the inspiratory inward or outward currents, at least five consecutive inspiratory bursts during control or drug application were low-pass-filtered at 5 Hz with an eight-pole Bessel filter and averaged for comparison, and the values during drug application were expressed as the ratios of control values (set as 1). When only two groups of results were statistically compared, paired or independent *t*-test was used when appropriate. When more than two groups of results were compared, generally one-way analysis of variance (ANOVA) followed by Bonferroni correction was applied, and additional paired *t*-test was used when appropriate. The results are normally presented as means ± SE, and the number of neurons or slices is expressed as “n”. The criterion for statistical significance was set at *p* < 0.05.

## Results

### AVP Depolarized AVPNs and Significantly Increased Their Spontaneous Firing Rate

**Figure 1:**
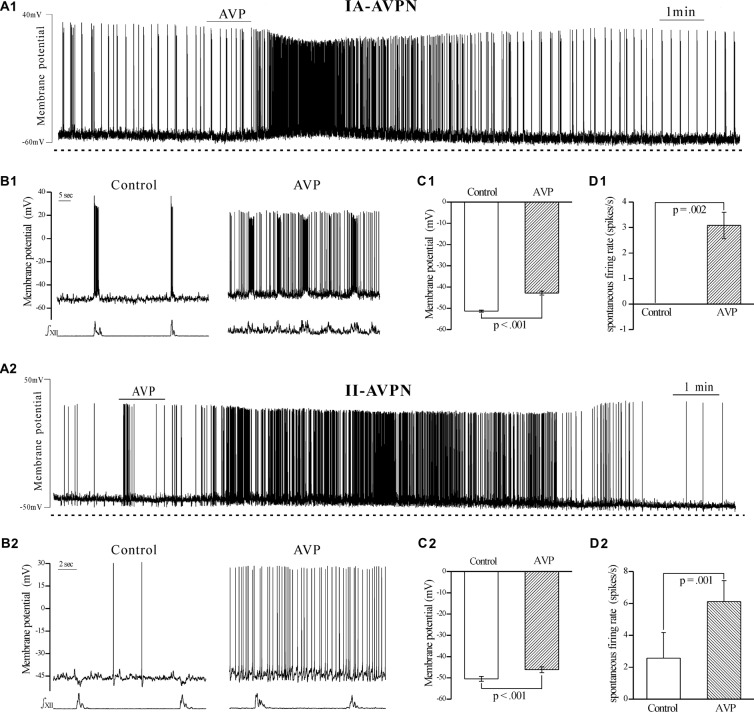
**Arginine vasopressin (AVP) significantly depolarized both types of airway vagal preganglionic neurons (AVPNs) and significantly increased their firing rate during inspiratory intervals. (A1)** Recording of a representative inspiratory-activated (IA)-AVPN. Note that AVP (100 nmol L^−1^) caused depolarization, increased the firing rate and decreased the amplitude of action potentials. **(B1)** Selected traces (upper trace) captured from **(A1)** are presented in an enlarged time-scale along with the simultaneous hypoglossal activity (lower trace), showing the firing during control (left) and AVP application (right). Please note that the IA-AVPN had no spontaneous firings during inspiratory intervals under the control condition but fired continuously in the presence of AVP. **(A2)** Similarly, AVP (100 nmol L^−1^) caused depolarization and increased the firing rate in a representative inspiratory-inhibited (II)-AVPN. **(B2)** Selected traces captured from **(A2)** are presented in an enlarged time-scale, showing that the sporadic action potentials during control recording became almost continuous after application of AVP, and the rhythmic inspiratory hyperpolarization became less pronounced. **(C1,C2)** Bar graphs showing the membrane potential in average before and during AVP application in IA-AVPNs (**C1**, *n* = 6) and II-AVPNs (**C2**, *n* = 6). Note the AVP-induced significant change. **(D1,D2)** Bar graphs showing the spontaneous firing rate in average in IA-AVPNs (**D1**, *n* = 6) and II-AVPNs (**D2**, *n* = 6). Note the AVP-induced significant increase. *P* values were obtained from paired *t*-test. ∫XII, integrated hypoglossal bursts.

AVP (100 nmol L^−1^) prominently depolarized both types of the AVPNs. The maximal depolarization ranged from 6 mV to 11 mV (mean 9 ± 2 mV, *n* = 6) in IA-AVPNs and 3 mV to 6 mV (mean 4 ± 1 mV, *n* = 6) in II-AVPNs. In both types of AVPNs, AVP significantly elevated the membrane potential to more positive levels (Figures 1A1–C1, 1A1–C1, A2–C2), significantly increased the spontaneous firing rate (Figures 1D1,D2), and slightly decreased the amplitude of action potentials. In addition, the frequency of the rhythmic hypoglossal bursts, as well as the synchronous inspiratory bursts in IA-AVPN and the inspiratory hyperpolarization in II-AVPN, was significantly increased, from 4.8 ± 2.1 bursts min^−1^ of control to 8.3 ± 2.5 bursts min^−1^ maximally during (after) application of AVP (*P* < 0.001, *n* = 14). In II-AVPNs, notably, the inspiratory hyperpolarization became less identifiable after AVP application (Figures 1A2,B2). The AVP-induced effects started within 2 min after application of AVP, lasted for 8–10 min, and were reversible.

### AVP Increased the Frequency of sEPSCs in Both Types of AVPNs, Augmented the Inspiratory Inward Currents in IA-AVPNs, but Suppressed the Inspiratory Outward Currents in II-AVPNs

**Figure 2:**
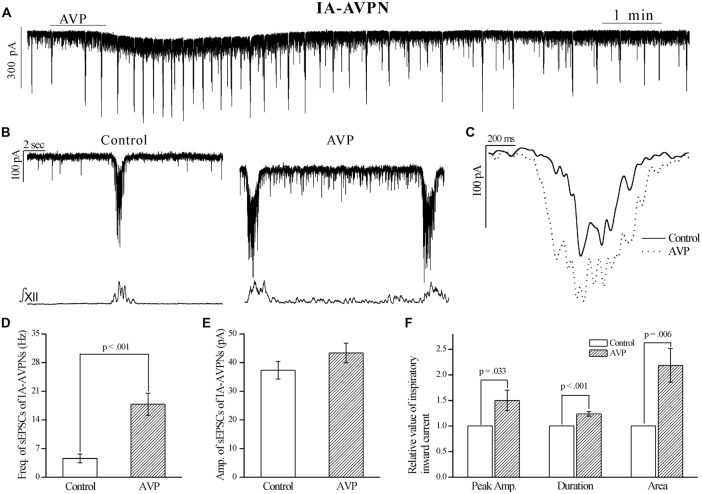
**AVP significantly increased the frequency of spontaneous excitatory postsynaptic currents (sEPSCs) and enlarged the rhythmic inspiratory inward currents in IA-AVPNs. (A)** Recording of a representative IA-AVPN under voltage clamp, showing that AVP (100 nmol L^−1^) caused a slow inward current and increased the frequency of the rhythmic inspiratory inward currents. **(B)** Selected traces captured from **(A)** are presented in enlarged time-scale along with the simultaneous hypoglossal activity, showing that AVP increased the frequency of sEPSCs during inspiratory intervals. **(C)** Comparison of the representative inspiratory inward current envelopes during control and AVP application, showing the AVP-induced changes. **(D,E)** Bar graphs for the frequency **(D)** and amplitude **(E)** of sEPSCs in averages (*n* = 10). Please note that the frequency but not amplitude of sEPSCs was significantly increased by AVP. **(F)** Bar graphs for the indices of inspiratory inward currents, showing the AVP-induced significantly increases in the peak amplitude, duration and area (*n* = 10). Freq., frequency; Amp., amplitude.

**Figure 3:**
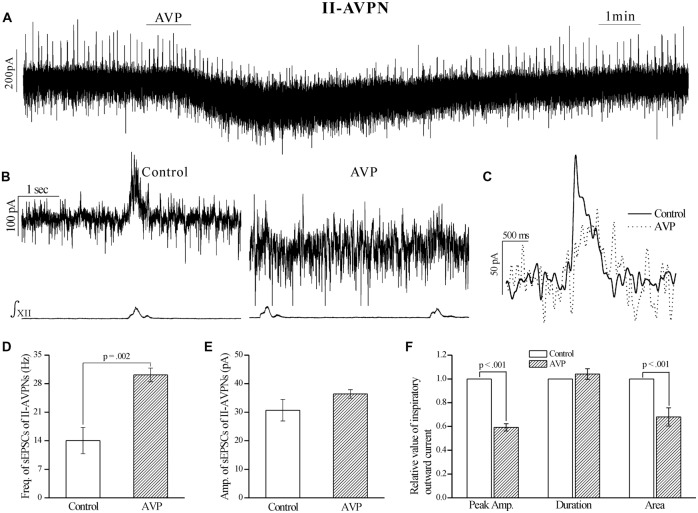
**AVP significantly increased the frequency of sEPSCs, but attenuated the rhythmic inspiratory outward currents in II-AVPNs.** **(A)** Recording of a representative II-AVPN under voltage clamp, showing that AVP (100 nmol L^−1^) caused a slow inward current and a distinct inhibition of the inspiratory outward currents.** (B)** Selected traces captured from **(A)** are presented in an enlarged time-scale, along with the simultaneous hypoglossal activity, showing the increase in the frequency of sEPSCs and the reduction of inspiratory outward currents. **(C)** Comparison of the representative inspiratory outward current envelopes during control and AVP application, showing the AVP-induced changes. **(D,E)** Bar graphs for the frequency **(D)** and amplitude **(E)** of sEPSCs in averages (*n* = 6). **(F)** Bar graphs for the indices of inspiratory outward currents (*n* = 6), showing the AVP-induced changes in the peak amplitude and area.

AVP (100 nmol L^−1^) significantly increased the frequency of sEPSCs, in both IA-AVPNs (Figures 2A,B,D) and II-AVPNs (Figures 3A,B,D), but did not significantly alter the amplitude of sEPSCs in either type of AVPNs (Figures 2E, 3E). And in IA-AVPNs, AVP significantly enhanced the inspiratory inward currents with respect to the peak amplitude, duration and area (Figures 2C,F); whereas in II-AVPNs, AVP significantly suppressed the peak amplitude and area of the inspiratory outward currents (Figures 3C,F). Additionally, AVP caused a slow inward current in both types of AVPNs (Figures 2A, 3A), of which the maximal amplitude ranged from 40 pA to 110 pA in IA-AVPNs (mean 73 ± 8 pA, *n* = 9) and 31–101 pA in II-AVPNs (mean 64 ± 11 pA, *n* = 6), respectively. At the end of the experiments, all of the excitatory inward currents, no matter spontaneous or phasic inspiratory, were eliminated by 50 μmol L^−1^ CNQX.

### The AVP-Induced Slow Inward Current, Frequency Increase of sEPSCs and the Changes in Phasic Inspiratory Currents in Both Types of AVPNs were Prevented by SR49059, but Unaffected by SSR149415

**Figure 4:**
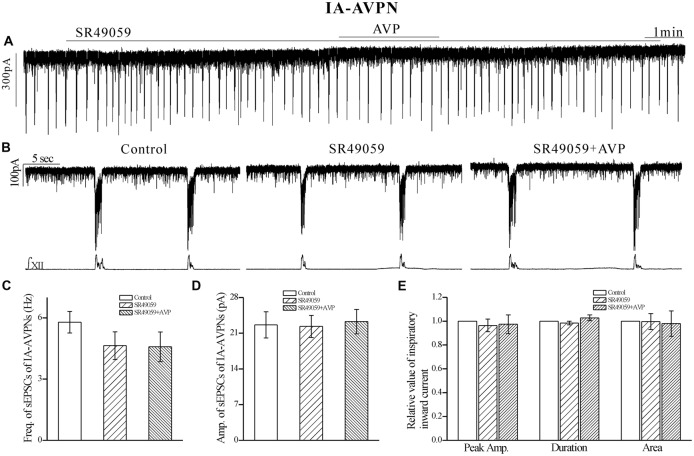
**The AVP-induced changes in sEPSCs and inspiratory inward currents in** **IA-AVPNs were prevented by SR49059, a selective antagonist of V_1a_ receptors.** **(A)** Recording of a representative inspiratory-activated (IA)-AVPN, showing that after pretreatment with SR49059 (20 μmol L^−1^), AVP (100 nmol L^−1^) caused little changes in the baseline current, sEPSCs and inspiratory inward currents. Note that SR49059 itself had little effects. **(B)** Selected traces captured from **(A)** are presented in an enlarged time-scale, along with the simultaneous hypoglossal activity. **(C–E)** Summarized data for the frequency **(C)** and amplitude **(D)** of sEPSCs in average (*n* = 6), and for the indices of inspiratory inward currents (**E**, *n* = 6).

**Figure 5:**
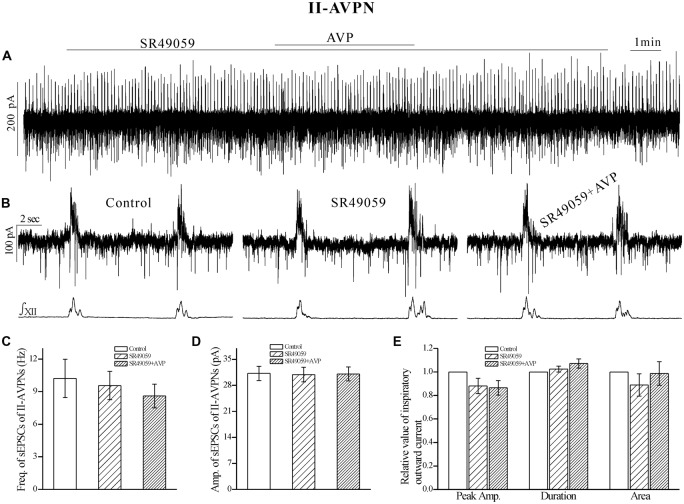
**The AVP-induced changes in sEPSCs and inspiratory outward currents in II-AVPNs were prevented by SR49059.** **(A)** Recording of a representative II-AVPN, showing that after pretreatment with SR49059 (20 μmol L^−1^), AVP (100 nmol L^−1^) caused little changes in the baseline current, sEPSCs and inspiratory outward currents. **(B)** Selected traces captured from **(A)** are presented in an enlarged time-scale, along with the simultaneous hypoglossal activity. **(C–E)** Summarized data for the frequency** (C)** and amplitude **(D)** of sEPSCs in average (*n* = 8), and for the indices of inspiratory outward currents (**E**, *n* = 6).

After pretreatment with SR49059 (20 μmol L^−1^), an antagonist of V_1a_ receptors, AVP no longer caused a noticeable slow inward current, and did not cause significant changes in respects of sEPSCs and phasic inspiratory currents in either IA-AVPNs (Figure 4) or II-AVPNs (Figure 5). In addition, SR49059 itself had no effect on either AVPNs or the hypoglossal activity.

**Figure 6:**
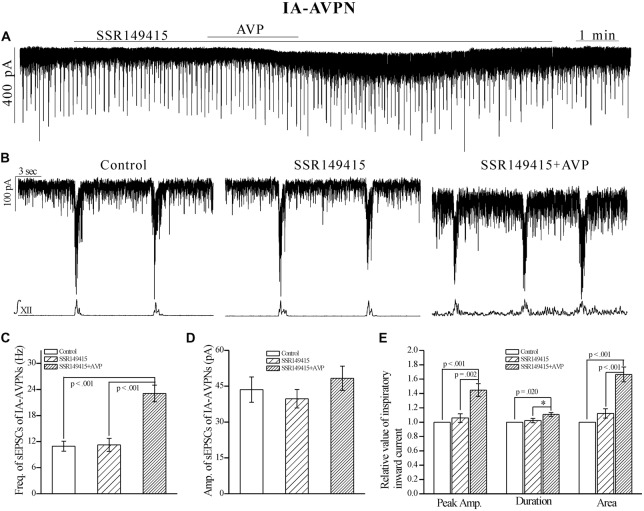
**The AVP-induced changes in sEPSCs and inspiratory inward currents in IA-AVPNs were unaffected by SSR149415, a selective antagonist of V_1b_ receptors. (A)** Recording of a representative IA-AVPN, showing that after pretreatment with SSR149415 (10 μmol L^−1^), AVP (100 nmol L^−1^) caused similar responses as it did without SSR149415. **(B)** Selected traces captured from **(A)** are shown in an enlarged time-scale, along with the simultaneous hypoglossal activity. **(C–E)** Summarized data for the frequency **(C)** and amplitude **(D)** of sEPSCs in average (*n* = 6), and for the indices of inspiratory inward currents (**E**, *n* = 6). *p < 0.05, obtained from additional paired *t*-test.

**Figure 7:**
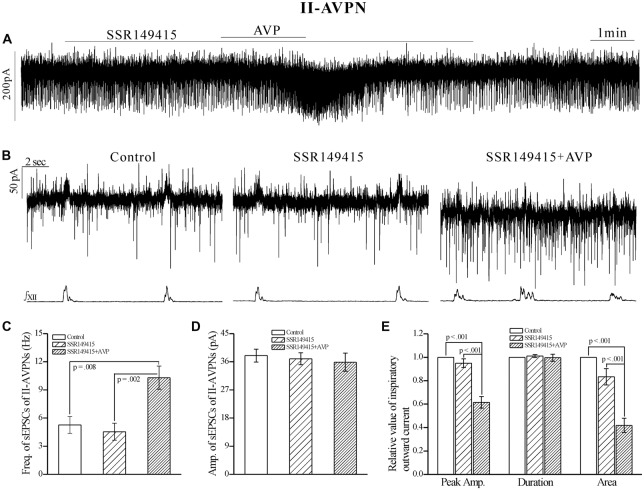
**The AVP-induced responses in II-AVPNs were unaffected by SSR149415.** **(A)** Recording of a representative II-AVPN, showing that after pretreatment with SSR149415 (10 μmol L^−1^), AVP (100 nmol L^−1^) caused similar responses as it did without SSR149415. **(B)** Selected traces captured from **(A)** are shown in an enlarged time-scale, along with the simultaneous hypoglossal activity. **(C–E)** Summarized data for the frequency** (C)** and amplitude **(D)** of sEPSCs in average (*n* = 7), and for the indices of inspiratory outward currents (**E**, *n* = 6).

In contrast, SSR149415 (10 μmol L^−1^), an antagonist of V_1b_ receptors, failed to block the effects of AVP. After pretreatment with SSR149415, AVP still caused a significant frequency increase of sEPSCs in both IA-AVPNs (Figures 6C,D) and II-AVPNs (Figures 7C,D), and induced a noticeable slow inward current (Figures 6A,B, 7A,B), of which the maximal amplitude ranged from 35 pA to 80 pA in IA-AVPNs (mean 57 ± 7 pA, *n* = 6) and 40–80 pA in II-AVPNs (mean 55 ± 7 pA, *n* = 6), respectively, and is not significantly different with that without the presence of SSR149415 (*P* = 0.26 in IA-AVPNs, *P* = 0.51 in II-AVPNs; independent *t*-test). And, like it did without SSR149415, AVP significantly augmented the inspiratory inward currents in IA-AVPNs (Figure 6E), and suppressed the inspiratory outward currents in II-AVPNs (Figure 7E). Similarly, SSR149415 itself had no effect on either AVPNs or the hypoglossal activity.

### AVP Significantly Increased the Frequency and Amplitude of GABAergic Spontaneous IPSCs (sIPSCs), All of Which were Prevented by SR49059

**Figure 8:**
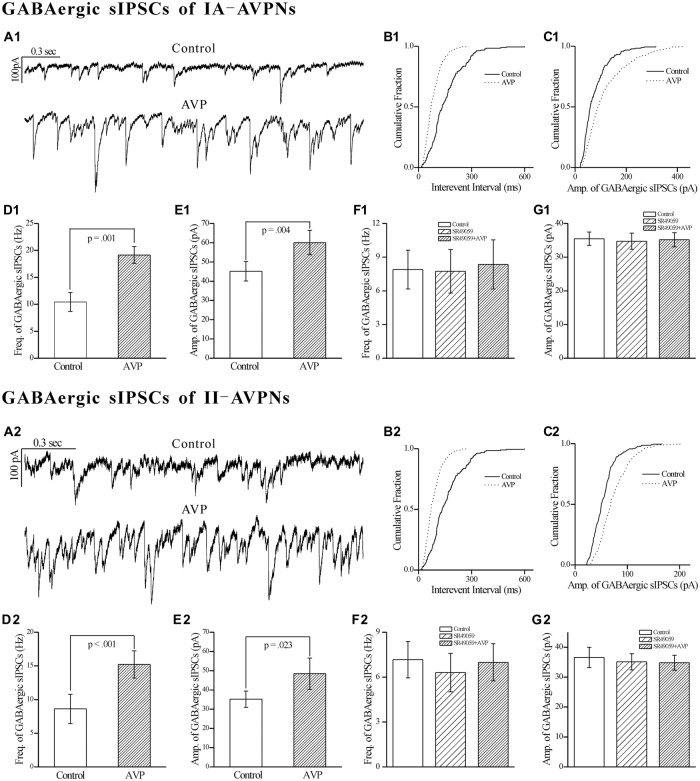
**AVP significantly increased the frequency and amplitude of GABAergic spontaneous inhibitory postsynaptic currents (sIPSCs) in both types of AVPNs, all of which were prevented by SR49059.** **(A1)** Representative recording segments of GABAergic sIPSCs from an IA-AVPN during control (upper panel) and AVP application (100 nM, lower panel), showing the AVP-induced changes in the frequency and amplitude. **(B1,C1)** Cumulative plots of the sIPSCs intervals **(B1)** and amplitude **(C1)** during control (continuous line) and AVP application (dotted line; Plotted from the same IA-AVPN in **A1**). Note that AVP caused a leftward shift in the interval distribution curve and a rightward shift in the amplitude distribution curve, indicating increases in the frequency and amplitude, respectively. **(D1,E1)** Bar graphs for the frequency and amplitude of GABAergic sIPSCs in average in IA-AVPNs (*n* = 6).** (F1,G1)** SR49059 (20 μmol L^−1^) prevented the AVP-induced increase in the frequency and amplitude (*n* = 6). **(A2–G2)** AVP caused similar changes in the GABAergic sIPSCs of II-AVPNs (*n* = 6) as in those of IA-AVPNs; and all of which were also prevented by SR49059 (*n* = 6).

In both types of the AVPNs, AVP (100 nmol L^−1^) significantly increased not only the frequency but also the amplitude of the pharmacologically isolated GABAergic spontaneous inhibitory postsynaptic currents (sIPSCs). Recording segments in Figure 8A1 showed the pharmacologically isolated GABAergic sIPSCs from a representative IA-AVPN during control and AVP application, for which the AVP-induced frequency increase is exhibited by a leftward shift of the cumulative distribution curve of inter-event intervals (Figure 8B1), and the AVP-induced amplitude increase by a rightward shift of the distribution curve of amplitude (Figure 8C1). Summarized (averaged) data for the frequency and amplitude of GABAergic sIPSCs in IA-AVPNs were shown in Figures 8D1,E1. Similar set of data from individual and multiple II-AVPNs were shown in Figures 8A2–E2.

All of the effects of AVP on GABAergic sIPSCs were prevented by pre-application of SR49059 (20 μmol L^−1^), in both IA-AVPNs (Figures 8F1,G1) and II-AVPNs (Figures 8F2,G2).

### AVP Significantly Increased the Frequency and Amplitude of Glycinergic sIPSCs in Both Types of AVPNs, All of Which were Blocked by SR49059

**Figure 9:**
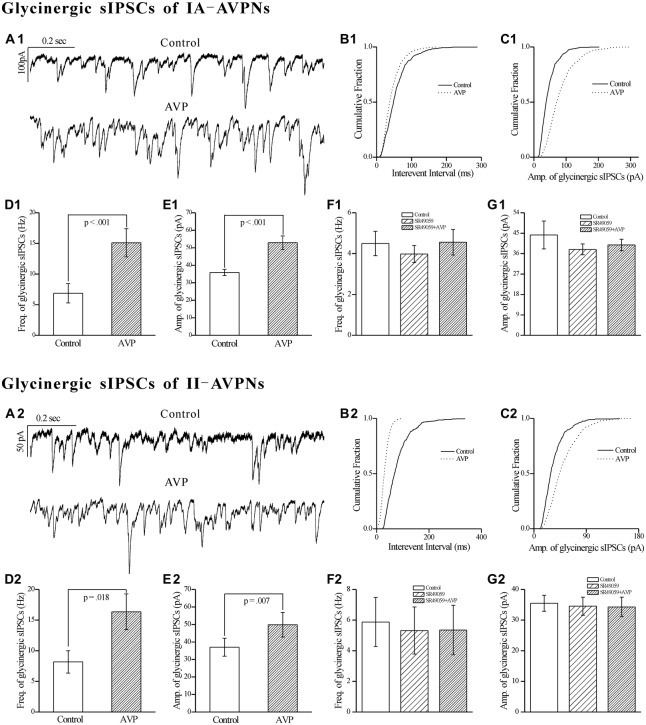
**AVP significantly increased the frequency and amplitude of glycinergic sIPSCs in both types of AVPNs, all of which were prevented by SR49059.** **(A1)** Representative traces of glycinergic sIPSCs from an IA-AVPN manifested the details during control (upper panel) and AVP application (100 nmol L^−1^, lower panel), showing the AVP-induced changes in the frequency and amplitude. **(B1,C1)** Cumulative plots of the sIPSCs intervals **(B1)** and amplitude **(C1)** during control (continuous line) and AVP application (dotted line; plotted from the same II-AVPN in **A1**). Note that AVP caused a leftward shift in the interval distribution curve and a rightward shift in the amplitude distribution curve, indicating increases in the frequency and amplitude, respectively. **(D1,E1)** Bar graphs for the frequency and amplitude of glycinergic sIPSCs in average in IA-AVPNs (*n* = 8). **(F1,G1)** SR49059 (20 μmol L^−1^) prevented the AVP-induced increase in the frequency and amplitude (*n* = 6). **(A2–G2)** AVP caused similar changes in the glycinergic sIPSCs of II-AVPNs as in those of IA-AVPNs (*n* = 6), and all of which were also prevented by SR49059 (*n* = 6).

Similar as it did on GABAergic sIPSCs, AVP (100 nmol L^−1^) significantly increased the frequency as well as the amplitude of the pharmacologically isolated glycinergic sIPSCs, in both IA-AVPNs (Figures 9A1–E1) and II-AVPNs (Figures 9A2–E2). Pre-application of SR49059 also abolished the effects of AVP on glycinergic sIPSCs in both IA-AVPNs (Figures 9F1,G1) and II-AVPNs (Figures 9F2,G2).

### AVP had no Effect on mEPSCs, mIPSCs and Membrane Input Resistance

After pre-treatment with TTX (1 μmol L^−1^), AVP (100 nmol L^−1^) did not cause significant changes in mEPSCs and mIPSCs, in either the frequency or amplitude, and in either type of AVPNs (data not shown). AVP failed to induce a significant change of the membrane input resistance, in either IA-AVPNs or II-AVPNs (data not shown). In the majority (12/15) of AVPNs, AVP no longer caused a measurable change in the baseline current; however it still elicited a slight slow inward current in a minority (3/15) of them, of which the maximal amplitude ranged from 21 pA to 36 pA.

## Discussion

This study has the following findings: (1) AVP depolarized both types of AVPNs and significantly increased the firing rate during inspiratory intervals; (2) AVP significantly increased the frequency of sEPSCs and caused a slow inward current in both types of AVPNs; (3) AVP significantly increased not only the frequency but also the amplitude of GABAergic and glycinergic sIPSCs in both types of AVPNs; (4) AVP significantly increased the intensity of the phase-locked inspiratory inward currents in respects of peak amplitude, duration and area in IA-AVPNs, but suppressed that of the inspiratory outward currents in II-AVPNs; (5) The AVP-induced changes were prevented by SR49059, an antagonist of V_1a_ receptor, but not by SSR149415, an antagonist of V_1b_ receptor; and (6) AVP had no impact on mEPSCs, mIPSCs and membrane input resistance in either type of AVPNs.

In the present study, AVP significantly increased the frequency of sEPSCs, and significantly increased both the frequency and amplitude of sIPSCs in both types of the AVPNs. While in the pre-existence of TTX, AVP failed to alter either the frequency or amplitude of mEPSCs and mIPSCs. These results prompt that the AVP-induced enhancement of sEPSCs or sIPSCs is action potential-dependent, and tend to rule out an effect of AVP on the presynaptic terminals of AVPNs. Most likely, the enhancement occurs on the somatodendritic membrane of the excitatory or inhibitory neurons presynaptic to AVPNs. This study also found that in most IA- and II-AVPNs, after pretreatment with TTX, the AVP-elicited slow inward currents disappeared; and in neither type of AVPNs, did AVP significantly alter the membrane input resistance. These results suggest that in most AVPNs, AVP has hardly a postsynaptic effect; and, when TTX was absent, the AVP-induced slow inward current might be primarily, if not completely, due to the summation of the enhanced synaptic inputs. However, in a minority (3/15) AVPNs, AVP did cause a slow inward current in the preexistence of TTX. This result suggests that vasopressin V_1a_ receptors, even truly expressed in AVPNs, the density might vary markedly among individual neurons. As a limitation, this study does not supply further immunohistological evidence regarding the expression of vasopressin V_1a_ receptors in AVPNs.

AVP significantly facilitated both the sEPSCs and sIPSCs of AVPNs. And, as is mentioned above, in most AVPNs, AVP showed little direct postsynaptic effect. However, AVP exclusively caused depolarization of AVPNs. These results suggest that in the integrated effect of AVP on AVPNs, the enhancement of excitatory inputs play an overwhelming role over the enhancement of inhibitory inputs. On the other hand, it should be born in mind that in our study, AVP was applied globally in the bath and it acted on the excitatory and inhibitory inputs of AVPNs without any temporal and spatial selectivity. However in intact CNS, vasopressinergic neurons are located in multiple brain nuclei (de Vries and Miller, 1998), and they might be activated by different types of stimuli and neuronal circuits. Therefore, it is possible that physiologically, the excitatory and inhibitory inputs of AVPNs are independently modulated by AVP; and AVP-induced extraordinary augmentation of individual type of inputs might cause dysregulation of airway vagal function.

AVP increased the frequency of the inspiratory inward currents in IA-AVPNs and that of the inspiratory outward currents in II-AVPNs. Given that the inspiratory EPSCs or IPSCs are rhythmically activated, the corresponding neurons presynaptic to AVPNs obviously receive the control from the inspiratory rhythmogenic neuronal network such as the pre-Bötzinger Complex (pre-BötC), or they themselves might right be the rhythmogenic neurons. A number of vasopressinergic fibers originating from the PVN do project to the pre-BötC, in which a subpopulation of neurons are immunoreactive for V_1a_ receptors; and microinjection of AVP into the pre-BötC caused a marked increase of respiratory rate (Kc et al., 2002). Thus it makes sense to infer that AVP might excite the excitatory rhythmogenic neurons in the pre-BötC and consequently increase both the frequency and the intensity of inspiratory inward currents.

Surprisingly, in II-AVPNs, AVP attenuated the inspiratory hyperpolarization under current clamp and significantly suppressed the peak amplitude and area of the inspiratory outward currents under voltage clamp. In view of the fact that AVP facilitated the pharmacologically isolated GABAergic and glycinergic sIPSCs of II-AVPNs, the suppression of the inspiratory outward currents by AVP is quite impressive. It must be emphasized that in our study, the inspiratory inward currents in IA-AVPNs were recorded with a holding voltage of −80 mV, at which the inhibitory synaptic currents mediated by GABA and glycine are minimized. As a result, the inspiratory inward currents in IA-AVPNs are not disturbed by inhibitory synaptic currents, and their enhancement by AVP might thus be fully revealed. While in II-AVPNs, the inspiratory outward currents were recorded at a holding voltage of −50 mV, at which the inhibitory synaptic currents are outward and the excitatory currents are inward. It should be born in mind that in II-AVPNs, excitatory inputs are also present during the inspiratory phase. Since AVP facilitated both the sEPSCs and the pharmacologically isolated sIPSCs of both types AVPNs, and, as is mentioned above, the AVP-induced facilitation of excitatory inputs seemingly surpasses that of inhibitory inputs, it is possible that in II-AVPNs, the excitatory inputs during the inspiratory phase were also enhanced by AVP. As a result, the inspiratory outward currents in II-AVPNs, whether altered by AVP independently or not, might be counteracted by an AVP-induced facilitation of the inward excitatory synaptic currents. In the present study, the sIPSCs and phasic inspiratory outward currents showed differential responses to AVP, which is somewhat consistent with the findings in our previous studies. One study found that thyrotropin-releasing hormone significantly suppressed the inspiratory outward currents of II-AVPNs while has no effect on their sIPSCs (Hou et al., 2012). Another found that an agonist of α_1_-adrenoceptors significantly enhanced the inspiratory outward currents of II-AVPNs but had no effect on their pharmacologically isolated GABAergic or glycinergic sIPSCs (Ge et al., 2015). Therefore, it is more possible that the inspiratory-related inhibitory inputs of II-AVPNs, which are primarily glycinergic (Hou et al., 2012), are somewhat different from the spontaneous inhibitory inputs in respect of the origin, and their inspiratory-related activity may be modulated distinctively.

So far, three types (V_1a_, V_1b_ and V_2_) of high-affinity receptors for AVP have been cloned and characterized by their primary structure, gene localization and functions. Among them, V_1a_ and V_1b_ receptors are found exclusively in the CNS (Koshimizu et al., 2012). And, in the brainstem, only V_1a_ receptors are abundantly expressed (Góźdź et al., 2003). SR49059, a selective antagonist of V_1a_ receptors, shows high binding affinity for V_1a_ receptors (rat: Ki = 2.2 nmol L^−1^) relative to V_1b_ and V_2_ receptors (Serradeil-Le Gal et al., 1993); while SSR149415 exhibits high affinity for V_1b_ receptors (rat: Ki = 3.7 nmol L^−1^; Serradeil-Le Gal et al., 2002). SR49059 alone completely blocked all of the AVP-induced effects, whereas SSR149415 failed to do so. These data strongly suggest that in this study, the AVP-induced changes in AVPNs are mediated by V_1a_ receptors. This conclusion is in accordance with the notion that the central actions of AVP are mainly, if not exclusively, mediated by V_1a_ receptors (Raggenbass, 2001).

Morphologically, AVP-containing fibers originating from the PVN project to the vicinity of AVPNs (Kc et al., 2006), and functionally, the results from our present study further certified that AVP do excite those AVPNs. AVP, as well as CRH (also excites the AVPNs, unpublished observation), is critically involved in the occurrence of psychological stress. It is likely that during the process of psychological stress, elevated central AVP leads to airway vagal excitation via activation of AVPNs, which subsequently induces or exacerbates asthma as is supposed by literatures (Ritz et al., 2010). Therefore, this study has provided a new insight for the contribution of stress in the induction or exacerbation of asthma. In fact, in the preliminary experiments of a prospective study, we have primarily validated that intracisternal application of SR49059 significantly attenuated the decrease of ventilatory function in ovalbumin-sensitized rats, via inhibition of airway vagal tone (unpublished data).

## Author Contributions

XY: acquisition and analysis of most data; preparation of the manuscript. XC, YG and DH: acquisition and analysis of some data; revision of the manuscript. YC and CX: improvement of experimental design and revision of the manuscript. JW: design of the experiments, interpretation of data and revision of the manuscript.

## Funding

This work was supported by the National Natural Science Foundation of China (NSFC) grants 81170019 and 81270060 to JW, and in part by the Natural Science Foundation of Shanghai (NSFS) grant 16ZR1403000 to YC.

## Conflict of Interest Statement

The authors declare that the research was conducted in the absence of any commercial or financial relationships that could be construed as a potential conflict of interest.

## Abbreviations

aCSF: artificial cerebral spinal fluid
AP5: D-2-amino-5-phosphonovalerate
AVP: arginine vasopressin
AVPNs: airway vagal preganglionic neurons
CNQX: 6-Cyano-7-nitroquinoxaline-2,3-dione
CNS: central nervous system
CRH: corticotropin-releasing hormone
CSF: cerebrospinal fluid
DMNV: dorsal motor nucleus of the vagus
eNA: the external formation of the nucleus ambiguus
HPA: hypothalamic-pituitary-adrenocortical
IA-AVPNs: inspiratory-activated airway vagal preganglionic neurons
II-AVPNs: inspiratory-inhibited airway vagal preganglionic neurons
IPSCs: inhibitory postsynaptic currents
mEPSCs: miniature excitatory postsynaptic currents
mIPSCs: miniature inhibitory postsynaptic currents
NA: nucleus ambiguus
pre-BötC: pre-Bötzinger Complex
PVN: paraventricular nucleus
sEPSCs: spontaneous excitatory postsynaptic currents
sIPSCs: spontaneous inhibitory postsynaptic currents
TTX: tetrodotoxin.
